# Real-time PCR assays for hepatitis B virus DNA quantification may require two different targets

**DOI:** 10.1186/s12985-017-0759-8

**Published:** 2017-05-12

**Authors:** Chao Liu, Le Chang, Tingting Jia, Fei Guo, Lu Zhang, Huimin Ji, Junpeng Zhao, Lunan Wang

**Affiliations:** 1 National Center for Clinical Laboratories, Beijing Hospital, National Center of Gerontology, No1 Dahua Road, Dongdan, Beijing, 100730 People’s Republic of China; 2 Beijing Engineering Research Center of Laboratory Medicine, Beijing Hospital, Beijing, People’s Republic of China; 3 Graduate School, Peking Union Medical College, Chinese Academy of Medical Sciences, Beijing, People’s Republic of China; 4 Department of Clinical Laboratory, Beijing Chaoyang Hospital, Capital Medical University, Beijing, People’s Republic of China

**Keywords:** Hepatitis B virus, Quantification, COBAS TaqMan HBV Test version 2, Duplex real-time PCR assays, Mutation

## Abstract

**Background:**

Quantification Hepatitis B virus (HBV) DNA plays a critical role in the management of chronic HBV infections. However, HBV is a DNA virus with high levels of genetic variation, and drug-resistant mutations have emerged with the use of antiviral drugs. If a mutation caused a sequence mismatched in the primer or probe of a commercial DNA quantification kit, this would lead to an underestimation of the viral load of the sample. The aim of this study was to determine whether commercial kits, which use only one pair of primers and a single probe, accurately quantify the HBV DNA levels and to develop an improved duplex real-time PCR assay.

**Methods:**

We developed a new duplex real-time PCR assay that used two pairs of primers and two probes based on the conserved S and C regions of the HBV genome. We performed HBV DNA quantitative detection of HBV samples and compared the results of our duplex real-time PCR assays with the COBAS TaqMan HBV Test version 2 and Daan real-time PCR assays. The target region of the discordant sample was amplified, sequenced, and validated using plasmid.

**Results:**

The results of the duplex real-time PCR were in good accordance with the commercial COBAS TaqMan HBV Test version 2 and Daan real-time PCR assays. We showed that two samples from Chinese HBV infections underestimated viral loads when quantified by the Roche kit because of a mismatch between the viral sequence and the reverse primer of the Roche kit. The HBV DNA levels of six samples were undervalued by duplex real-time PCR assays of the C region because of mutations in the primer of C region.

**Conclusions:**

We developed a new duplex real-time PCR assay, and the results of this assay were similar to the results of commercial kits. The HBV DNA level could be undervalued when using the COBAS TaqMan HBV Test version 2 for Chinese HBV infections owing to a mismatch with the primer/probe. A duplex real-time PCR assay based on the S and C regions could solve this problem to some extent.

## Background

Hepatitis B virus (HBV) is prevalent worldwide, with an estimated 400 million people infected with HBV globally [1]. About 350 million people have chronic HBV infections [2]. Annually, HBV-related cirrhosis and hepatocellular carcinoma account for more than 750,000 deaths worldwide [3]. HBV infection has a heavy economic and social burden for both HBV patients and society in general.

In addition to tests for HBV serological markers, which include HBsAg, anti-HBsAg, HBeAg, anti-HBeAg and anti-HBc, and serum alanine transaminase (ALT) and aspartate transaminase (AST) tests, quantification of HBV DNA, which reflects the viral load, could also aid in measurement and management of HBV infections. HBV DNA levels change during different phases of infection in chronically HBV infected individuals [4]. HBV DNA measurements play a critical role in determining the phase of infection, deciding the treatment, and detecting responses to the antiviral therapy [2]. According to the guidelines for the prevention, care, and treatment with persons with chronic hepatitis B virus from the World Health Organization and China, HBV DNA quantification is recommended in the treatment of chronic HBV infections [5, 6].

Hepatitis B virus has a highly variable DNA genome and is divided into genotypes A– H based on the 8% sequence variations in the S region [7]. These genotypes can be further differentiated into subgenotypes [8]. Because HBV replicates its DNA genome through reverse transcription of pregenomic RNA and its DNA polymerase lacks proofreading activity, the mutation rate is higher than that in most of the other DNA viruses [8–10]. Long-term treatment with antiviral drugs such as lamivudine and adefovir can also lead to mutations in the HBV genome. The typical mutation caused by lamivudine is a mutation in the YMDD motif of the polymerase [11]. In addition to the common drug resistance mutations, other variations, both characterized and uncharacterized, may occur due to HBV treatment.

Unlike traditional PCR, real-time PCR, with its increased accuracy, wider linear range, and reproducibility, is widely used for the quantitative detection of HBV DNA [12, 13]. Currently, most HBV DNA quantification reagents use one pair of primers and a single probe for a given HBV genotype test. If HBV genetic variations exist in these primer or probe regions, the actual viral load of HBV will be underestimated by the assay. Mutations in the probe region of the COBAS Amplicor test caused by lamivudine led to the underestimation of the HBV DNA level of a chronic hepatitis patient [14].

Human immunodeficiency virus (HIV) is a genetically diverse retrovirus, and several pairs of primers and probes were helpful for its nucleic acid test [15, 16]. Thus, two pairs of primers and probes may prevent the underestimation of HBV DNA levels caused by mutations in the primer or probe regions. A duplex real-time PCR method for HBV DNA detection has been described; however, the advantages of this method for the quantitative determination of HBV DNA have not been demonstrated [17].

China has a moderately high prevalence of HBV infections, and the positive rate for HBsAg was 7.2% according to an epidemiological survey in conducted in 2006 [18]. Additionally, HBV is a highly genetically varied DNA virus and there are a number of drug-resistant mutants. We speculate that it is necessary to quantify HBV DNA levels using at least two pairs of primers and probes. The aim of the study was to: 1) to validate feasibility of a duplex real-time PCR method for HBV DNA quantification; and 2) to confirm the necessity of two pairs of primers and probes for HBV DNA quantitation.

## Methods

### Primers and probes selection for the duplex real-time PCR

One thousand HBV complete sequences from Chinese patients were downloaded from the NCBI database, which include genotypes A, B, C, D, and G and were aligned by the DNAman software. Primers and probes were designed by Primer 5 to target the conserved sequences of the S and C regions of the HBV genome. Primers were selected by testing using EvaGreen, and primers with significant dimerization were removed. Probes with lower count (Ct) values were chosen for further experiments (data not shown). The primers and probes are listed in Table 1.

**Table 1:** The primers and probe sequences for the S and C regions

Primer and Probe	Sequence(5′-3′)
S-F	GATGTGTCTGCGGCGTTTTA
S-R	GCAACATACCTTGATAGTCCAGAAGAA
S-P	Vic-CCTCTICATCCTGCTGCTATGCCTCA-BHQ1
C-F	TTCCGGAAACTACTGTTGTTAGAC
C-R	ATTGAGATTCCCGAGATTGAGA
C-P	Fam-CCCTAGAAGAAGAACTCCCTCGCCTC-BHQ1

### Standard materials

Plasma from a patient with a high-titer viral load was serially diluted 1:10 seven times. The plasma serial dilutions were quantified using the COBAS TaqMan HBV Test version 2 (Cobas, Switzerland). The quantified values were evaluated using the World Health Organization (WHO) Second International Standard for HBV DNA (National Institute for Biological Standards and Control, NIBSC; code 96/798, UK).

### DNA extraction and detection

Template DNA was extracted from 400 μl plasma with an automatic ExiPrep Dx Viral DNA kit (Bioneer, Korea) on an ExiPrep 16 Dx according to the manufacturer’s instructions. We added the primers S-F (concentration: 0.8 μmol/μl), S-R (concentration: 0.8 μmol/μl), S-P (concentration: 0.2 μmol/μl), C-F (concentration: 0.3 μmol/μl), C-R (concentration: 0.3 μmol/μl), and C-P (concentration: 0.1 μmol/μl) into same the tube to target both the S and C regions of the HBV genome. Baorui master mix (5×; baorui, Zhuhai, China) was used for PCR reactions with the SLAN-96S Real-Time PCR System (Slan, Shanghai, China). The qPCR volume was 100 μl with 74 μl DNA template. The PCR program was: 50 °C for 2 min, 95 °C for 1 min, 95 °C for 5 s, 55 °C for 40 s, and then 60 cycles of 25 °C for 1 min.

### Linearity

Serially diluted standard samples were prepared with concentrations from 2 × 10^8^ to 2 × 10^1^ IU/ml. Each concentration was tested in triplicate.

### Limit of detection (LOD) and specificity

The WHO Second International Standard for HBV DNA was diluted to 100, 50, 20, 10, 5, and 2.5 IU/ml. Each concentration was tested 20 times. Probit analysis was used to determine the LOD of the assay. Fifty samples of HBV-negative serum or plasma were tested by the assay to analyze specificity.

### Patients and plasma

Group A consisted of 104 plasma samples obtained from patients at the 302 Military Hospital of China and group B consisted of 59 plasma samples obtained from patients at Beijing ChaoYang Hospital between July 2016 and August 2016. Group C was 75 HBV DNA positive plasma samples obtained from blood banks in China. HBV DNA in Group A and Group C samples was quantitatively analyzed using the COBAS TaqMan HBV Test version 2 (CTM, v2), while samples from Group B were analyzed with the Daan real-time PCR assay. Both assays were performed according to the manufacturer’s instructions. All samples were stored at −30 °C. The institutional review board of the National Center for Clinical Laboratories approved this study. The methods in the study are in accordance with the guidelines of the Declaration of Helsinki. Written informed consent was obtained from all subjects participating in this study.

### Comparison of quantitative results for HBV DNA, sequencing, and plasmid construction and validation

The samples with quantitative differences larger than 1 log_10_ IU/ml between the in-house method and the CTM, v2 assay were used to amplify the full-length or pre-Core and Core regions of HBV, according to the methods described by Günther S and Lieven Stuyver, respectively [19, 20]. The HBV PCR products from these samples were cloned into a T vector and then sequenced by Sangon Biotech Co., Ltd. (Shanghai). The concentrations of plasmid DNA were measured using a Meriton SMA6000 (Meriton, USA) and converted to copies/ml and IU/ml (1 IU = 5.82 copies). The plasmids were quantitatively measured in triplicate by the CTM, v2 assay; Daan real-time PCR assay; and the in-house method. The samples with differences in the quantitative results between the S and C regions using the in-house duplex PCR were amplified using the primers in Table 2 and sequenced. At the same time, four samples had quantitative results from the in-house assay that were similar with that of CTM, v2 assay and were also amplified and sequenced providing reference sequence controls. The alignment of these sequences was analyzed using DNAman software.

**Table 2:** The primers for amplification of the S and C regions

Primer	Sequence(5′-3′)
Sa-F	TCGTGTTACAGGCGGGGTTT
Sa-R	GGCACTAGTAAACTGAGCCA
Ca-F	CCTACTGTTCAAGCCTCCAA
Ca-R	AATGTCCTCCTGTAAATGAATGT

### Statistical analysis

Statistical analyses were performed using SPSS software version 19.0 and Graph Prism 5.0. Pearson’s correlation coefficients and linear regression were used for correlation analysis. Bland-Altman tests were used to assess the agreement of the quantitative results of HBV DNA between the CTM, v2 assay or the Daan test and the in-house method.

## Results

### LOD of the duplex real-time PCR assay

The sensitivity test, which was performed using 20 replicates of different concentrations (10^4^, 10^3^, 100, 50, 20, 10, 5, 2.5 IU/ml), showed that the lowest 100% positive concentration for the duplex real-time PCR assay was 20 IU/ml (Table 3). These results were further subjected to probit regression analysis. The LODs of the S region, the C region, and the duplex real-time PCR assay were 15.2 IU/ml (95% confidence interval, 12.2–23.4 IU/ml), 16.6 IU/ml (95% confidence interval, 12.9–26.1 IU/ml), and 11.9 IU/ml (95% confidence interval, 9.2–19.8 IU/ml), respectively.

**Table 3:** Limit of detection for the S and C regions using the duplex real-time PCR assay

HBV load(IU/ml)	S region		C region		Duplex	
	Positive results/total tests	Positive rate (%)	Positive results/total tests	Positive rate (%)	Positive results/total tests	Positive rate (%)
100	20/20	100	20/20	100	20/20	100
50	20/20	100	20/20	100	20/20	100
20	20/20	100	20/20	100	20/20	100
10	14/20	70	13/20	65	18/20	90
5	6/20	30	9/20	45	10/20	50
2.5	4/20	20	5/20	25	7/20	40

### Specificity

All 50 HBV-negative samples were determined by the duplex real-time PCR to be negative for HBV DNA demonstrating 100% specificity.

### Linearity

**Fig. 1:**
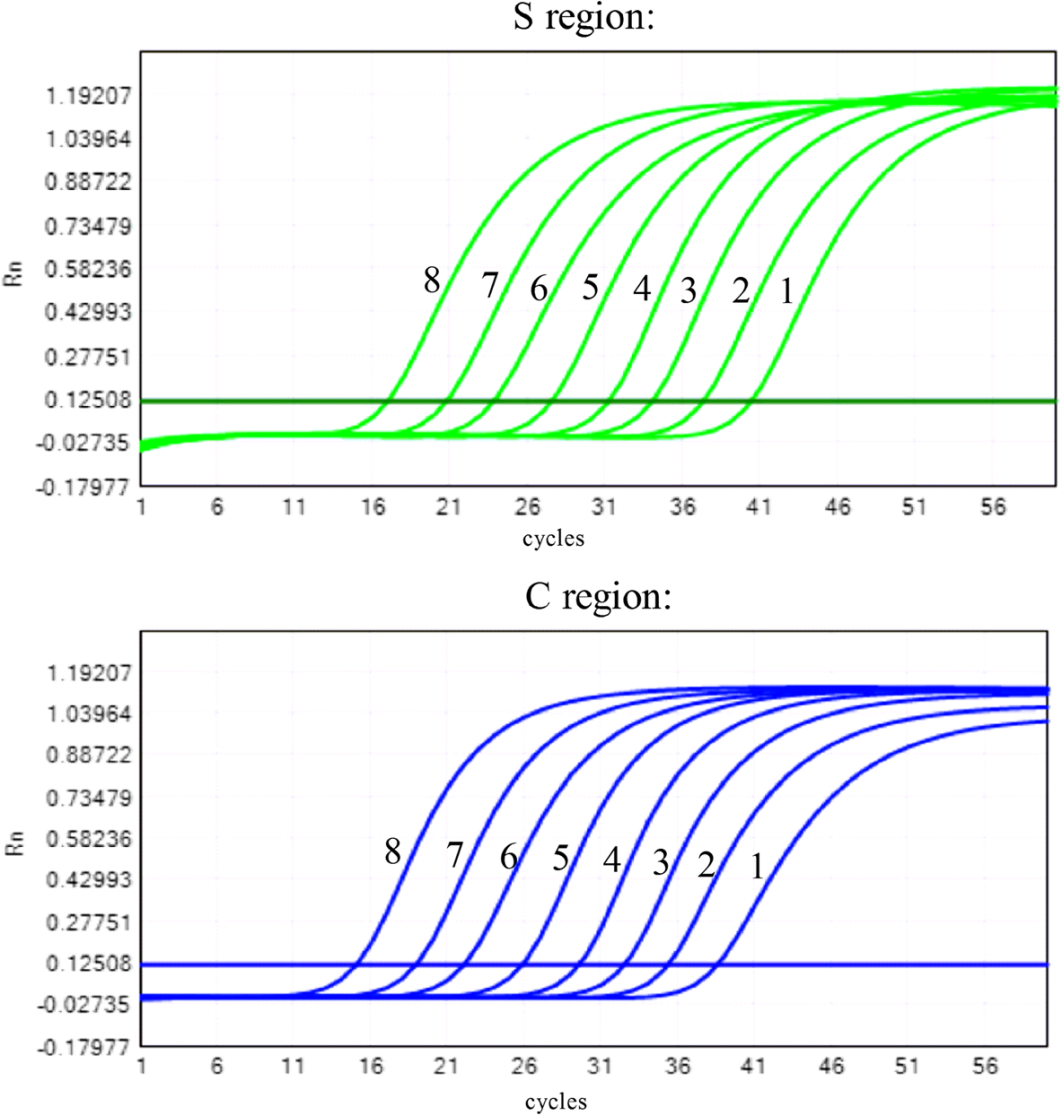
The amplification curves for the S and C regions using the duplex real-time PCR assay with diluted high-titer HBV samples (from 2.0 × 10^8^ to 2.0 × 10^1^ IU/ml)

Ten-fold dilutions of genotype B samples were used for the linearity test of the duplex real-time PCR. The results showed a good correlation. The linear regression equations for the S and C regions were Y = −3.39X + 45.39 (R^2^ = 99.9%) and Y = −3.43X + 43.93 (R^2^ = 99.5%), respectively (Fig. 1).

### Comparison of the viral loads determined by the duplex real-time PCR assay with the CTM, v2 assay

**Fig. 2:**
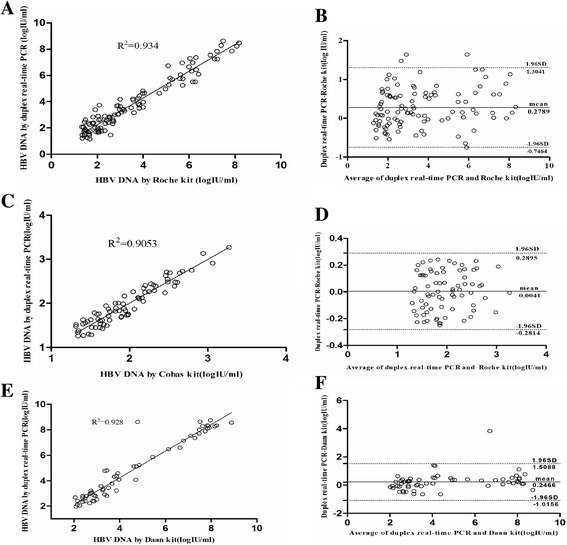
Correlation and Bland-Altman analysis of quantitative results of the duplex real-time PCR assay, Roche CAP/CTM v2, and Daan real-time PCR assay. **a** and **b** analysis of the quantitative results from the duplex real-time PCR assay and Roche CAP/CTM v2 with 104 HBV samples collected from the 302 Military Hospital of China. **c** and **d** analysis of the quantitative results of the duplex real-time PCR assay and Roche CAP/CTM v2 with 75 HBV samples collected from blood banks in China. **e** and **f** analysis of the quantitative results of the duplex real-time PCR and Daan real-time PCR assays with 59 HBV samples collected from the ChaoYang Hospital in China

A total of 104 HBV samples, which were collected from the 302 Military Hospital and 75 HBV samples collected from blood banks in China were quantitatively analyzed by the duplex real-time PCR assay. The viral loads of most of the HBV samples were similar for CTM v2.0 assay and the duplex assay (Fig. 2). However, there were 11 samples with viral loads determined by the duplex assay that were 1.0 log higher than that determined by the Roche diagnostics kit. At the same time, the samples from blood banks had similar results for the CTM, v2.0 assay and duplex real-time assays (Table 4).

**Table 4:** Patient samples that showed significantly different DNA quantification results using the Roche kit, the Daan kit, and the duplex real-time PCR assay

Sample no.	Age	Sex	Cobas	Daan	Duplex-S	Duplex-C
302-1	6	F	65.2	No	694	188
302-17	45	F	31.2	No	414	310.2
302-44	25	M	991	No	15010	15470
302-45	1	F	3.06E7	No	4.17E8	1.83E8
302-66	26	M	2.00E6	No	2.32E7	1.90E7
302-68	40	M	5.06E5	No	6.68E6	9.07E6
302-74	65	F	28.3	No	286	62.58
302-76	51	M	1.24E5	No	5.47E6	3.74E6
302-80	52	M	1.10E6	No	2.00E7	3.76E6
302-91	20	M	124	No	5437	488
302-102	45	M	81.9	No	2470	165
Chaoyang-9	62	F	No	6.14E4	1.35E6	1.01E6
Chaoyang-36	82	M	No	2.76E3	6.32E4	8.46E3
Chaoyang-53	26	F	No	2.24E3	5.83E4	1.93E4

*Only patients whose HBV DNA quantitative results from the duplex real-time PCR were at least 1.0 log_10_ higher than that using Roche kit or Daan kit are listed. (Unit: IU/ml)

### Comparison the viral load of the duplex real-time PCR assay with Daan real-time PCR assays

A total of 59 HBV samples, which were collected from ChaoYang Hospital, were quantitatively analyzed by the Daan kit and duplex real-time PCR assays. The HBV DNA viral load results were compared using correlation and Bland-Altman analysis. This analysis revealed a statistical correlation between the results of the Daan kit and the duplex real-time PCR (R^2^ = 0.923, *p* < 0.0001). A mean difference of 0.22 ± 0.67 log_10_ IU/ml (limits of agreement: from −1.08 to 1.53 log_10_ IU/ml) was observed between them (Fig. 2). Among the 59 samples, the viral load of 3 samples were determined by the duplex real-time PCR to be 1.0 log_10_ higher than the results of the Daan kit, with differences of 1.34, 1.36 and 1.41 log_10_ (Table 4).

### Comparison the viral load determined using the S and C regions with the duplex real-time PCR assay

**Fig. 3:**
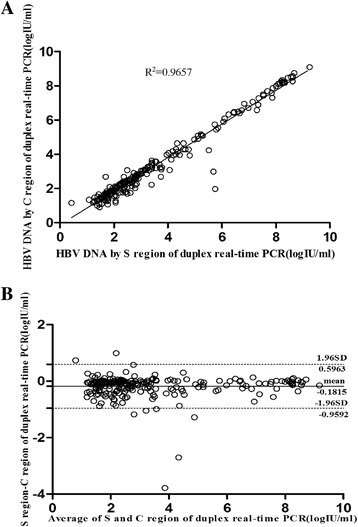
The correlation and bland-altman analysis of the quantitative results of S and C regions of duplex real-time PCR assays in 238 HBV samples. **a**: The correlation analysis of the quantitative results of S and C regions of duplex real-time PCR assays in 238 HBV samples. **b**: The bland-altman analysis of the quantitative results of S and C regions of duplex real-time PCR assays in 238 HBV samples

A total of 238 HBV samples, which were collected from the 302 Military Hospital, ChaoYang Hospital, and blood banks in China, were analyzed by the duplex real-time PCR. HBV DNA levels were further analyzed according to the S and C regions for the duplex real-time PCR. These results revealed a statistical correlation between the results from the S and C regions (R^2^ = 9657, *p* < 0.0001). A mean difference of −0.18 ± 0.40 log_10_ IU/ml (limits of agreement: from −0.96 to 0.60 log_10_ IU/ml). Among the 238 HBV samples, the S region results of 8 samples were more than 1.0 log_10_ IU/ml higher than that of the C region using the duplex real-time PCR (Fig. 3).

### Sequence alignment and variability of samples for which quantitative results of the S region were more than 1.0 log_10_ higher than those of the C region

**Fig. 4:**
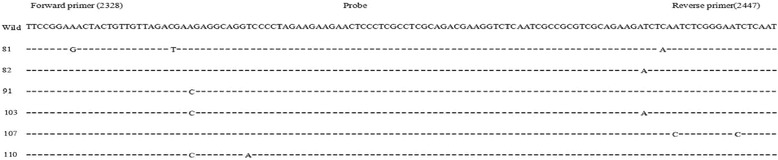
Alignment of C region primers using in the duplex real-time PCR assay with sequences of samples that had DNA quantitative results for the C region that were more than 1.0log_10_ IU/ml lower than that for the S region

Among the eight samples with quantitative results using the S region with the duplex real-time PCR greater by more than 1.0 log_10_ IU/ml than results using the C region, six samples were successfully amplified and sequenced. Mutations occurred in the forward and reverse primers in four samples; however, there were no mutations in the primer/probe region in another two samples, only mutations in the amplified region outside the primer/probe sequence (Fig. 4).

### Sequence alignment and variability of samples for which quantitative results by duplex real-time PCR were more than 1.0 log_10_ higher than those obtained by Roche kit

Among the 11 samples with quantitative results using Roche kit lower by more than 1.0 log_10_ IU/ml than that using the duplex real-time PCR, the full-length or pre-Core and Core region of only four samples were successfully amplified and sequenced. Another four samples with similar results using the Roche kit and the duplex real-time PCR were amplified and sequenced as control sequences. After alignment, we found several different sites in the 1827–1970 nt region, which is the target region of Roche kit. The different sites in the four samples are listed in Table 5.

**Table 5:** Mutations in samples that yielded lower DNA quantitative results (at least 1.0 log_10_ IU/ml) with the Roche kit than with the duplex real-time PCR assay

Sample. no.	Different sites
44	C1962T, T1963G
45	T1858C, G1915C, T1936C
66	G1915C,
76	G1899A, G1915A, G1937A, T1938A, T1961G, C1962G

### Validation using plasmids

The sequences of four samples with quantitative results using the duplex real-time PCR assay greater by more than 1.0 log_10_ IU/ml than results using the Roche kit were cloned into a T vector and sequenced. The sequences of another four samples with similar results for the Roche kit and the duplex real-time PCR were also cloned into a T vector. The concentrations of all plasmid were measured and converted to copies/ml and IU/ml. The plasmids at a concentration of 1.0^4^ IU/ml were quantitatively detected by the duplex real-time PCR and the Daan real-time PCR. Quantitative results showed two of the four samples had results lower by more than 1.0 log_10_ IU/ml compared to the other assays (Table 6).

**Table 6:** Validation of plasmids containing the HBV DNA sequences of samples with higher viral loads (at least 1.0 log_10_IU/ml) detected with the Roche kit was 1.0 log_10_IU/ml lower than that of duplex real-time PCR assay

Sample No.	Concentration^a^	Roche	Daan	Duplex-S	Duplex-C
Wild	1.0e4	1.79e4	1.01e4	1.7e4	1.5e4
302-44^b^	1.0e4	3.09e2	7.41e3	No	No
302-45	1.0e4	5.29e3	5.16e3	6.19e3	9.03e3
302-66	1.0e4	2.45e3	3.83e3	1.42e4	1.0e4
302-76^b^	1.0e4	2.36e2	5.37e3	1.27e4	1.49e4

^a^Calculated from the concentration of the plasmid

^b^The samples for which quantitative results by Roche were more than 1.0log_10_IU/ml than that of other assays (Unit: IU/ml)

## Discussion

Due to the effects of genetic variations on HBV DNA quantification, we developed a duplex real-time PCR assay using two pairs of primers and probes based on conserved sequences of the S and C regions of the HBV genome. In this study, we confirmed that the quantitative results of the S and C regions were similar, but that mutations in the amplified region led to an underestimation of the C region in four samples. At the same time, the results of the duplex real-time PCR were also in good agreement with that of the Roche and Daan kits; however, underestimation of DNA levels could be present in 11 samples using the Roche kit and 3 samples using the Daan kit. This study demonstrated that commercial kits, which contained only one pair of primers and a single probe, might underestimate HBV DNA levels in some HBV specimens. It also implied that at least two pairs of primers and two probes are necessary for HBV DNA quantification, and the duplex real-time PCR can be used for this application.

In our study, we chose the Roche kit, which is widely used for HBV DNA quantification around the world and the Daan kit, which is used widely in China for analysis. The Roche kit uses one pair of primers and a single probe for a specific sample. Our study demonstrated that the Roche and Daan kits could lead to underestimation of HBV DNA levels because of the variations in the amplified region. This implies there is a defect in kits with only one pair of primers and a single probe for HBV DNA quantification. At the same time, it also demonstrated the necessity of using two pairs of primers and two probes for the quantitative detection of HBV DNA, which is highly variable. In previous studies, it was demonstrated that several primers and probes were helpful for the nucleic acid test for HIV, which is a genetically variable retrovirus [15, 16]. In our study, we developed a duplex real-time PCR that used two pairs of primers and two probes, which were based on the S and C regions of HBV. It also showed good agreement with the Roche and Daan kits and demonstrated good correlation between results for the S and C regions and linearity, which confirms the feasibility of using the duplex real-time PCR.

On the other hand, there were several shortcomings of this study. First, the number of the samples used for the comparison of the Roche and Daan kits with the duplex real-time PCR was small. Second, the exact sequences of the primers and probes of the Roche and Daan kits were unknown. We could only perform alignment of the target region sequences of the underestimated and accordant samples and validate the sequence variation through construction of plasmids containing whole HBV DNA or an amplified region. Third, the duplex real-time PCR assay was not a validated kit and there was no internal control.

Previous studies have demonstrated that several commercial kits, which use only one pair of primers and a single probe, could not detect hepatitis C virus (HCV) samples whose sequences had mismatches with primers or probes [21, 22]. Real-time PCR assays using two primers and probes have been developed for HCV and HIV detection [23, 24]. HBV is a highly variable DNA virus and with many mutations that emerge with the use of antiviral drugs. It has been reported that the HBV DNA level of an patient who was put on long-term lamivudine therapy was underestimated by more than 1.5 log_10_ IU/ml by the COBAS Amplicor assay because of lamivudine resistance mutations in the probe region [14]. Sun et al. developed a duplex real-time PCR using two pairs of primers and two probes targeting the S region; however, the assay was only used to screen for HBV DNA, and it has not been demonstrated that mutations in probes or primers could affect the detection of HBV [17]. In this study, we developed a duplex real-time PCR assay to use for HBV DNA quantification. We confirmed that mutations in primers or probes could lead to the underestimation of HBV DNA levels in both the duplex real-time PCR assay and the Roche kit. The mutations in the forward and reverse primers targeting the C region in our in-house duplex real-time PCR led to the underestimation of DNA levels. The mutations T1938A, T1961G, and C1962G were also observed in the samples with HBV DNA levels determined by the Roche kit to be more than 1 log_10_ IU/ml lower than determined by the duplex real-time PCR, which is consistent with a previous study [25]. At the same time, we also found 3 out of 59 HBV samples were underestimated by the Daan kit. There are about one third of 350 million HBV carriers living in China. The natural polymorphisms in many HBV sample may be complex and drug resistance mutations, which may emerge with antiviral drug usage. Thus, the commercial kits using only one pair of primers and a single probe may underestimate the HBV DNA levels of samples that have mismatches with primers/probes. As several pairs of primers and probes are used to detect HCV and HIV, we developed a duplex real-time PCR assay to try to solve the underestimation problem. The duplex real-time PCR assay showed good linearity and agreement with the Roche and Daan kits. This demonstrated that a real-time PCR assay containing at least two pairs of primers and probes could avoid underestimation in HBV samples.

Accurate HBV DNA quantification plays a critical role in determining the phase of infection, deciding the treatment, and detecting responses to antiviral therapy [2]. We found that HBV DNA levels could be underestimated by commercial kits in Chinese HBV-infected patients. If the commercial kits used two or more pairs of primers and probes for HBV quantification, the probability of underestimating the DNA levels would significantly drop. At the same time, if manufacturers published the sequences of primers and probes in commercial kits, it allow for better determination of the problem when patient HBV DNA level underestimation occurred.

## Conclusions

In conclusion, we developed a new duplex real-time PCR assay for HBV DNA quantification and confirmed that this in-house assay was feasible. The HBV DNA levels of two Chinese patients were underestimated by the COBAS TaqMan HBV Test version 2 assay by more than 1.0 log_10_ IU/ml due to a mismatch in the reverse primer. The HBV DNA levels of six samples were also underestimated due to a mismatch in the primer used in this duplex real-time PCR assays. A duplex real-time PCR assay based on the S and C regions could solve this problem to some extent. These results indicate that accurate HBV DNA quantification may require at least two amplification targets.

## Abbreviations

ALT: Alanine transaminase
AST: Aspartate transaminase
CTM, v2: COBAS TaqMan HBV Test version 2
Daan kit: Daan real-time PCR assays
HBV: Hepatitis B virus
LOD: Limit of detection
Roche kit: COBAS TaqMan HBV Test version 2

## References

[1] World Health Organization (2011). Hepatitis B. Fact sheet no. 204.

[2] European Association For The Study Of The Liver (2012). EASL clinical practice guidelines: Management of chronic hepatitis B virus infection. J Hepatol.

[3] Fattovich G, Bortolotti F, Donato F (2008). Natural history of chronic hepatitis B: special emphasis on disease progression and prognostic factors. J Hepatol.

[4] Chevaliez S, Rodriguez C, Pawlotsky JM (2012). New virologic tools for management of chronic hepatitis B and C. Gastroenterology.

[5] WHO Guidelines Approved by the Guidelines Review Committee (2015). Guidelines for the prevention, care and treatment of persons with chronic hepatitis B infection.

[6] Hou JL, Lai W, Chinese Society of Hepatology, Chinese Medical Association, Chinese Society of Infectious Diseases, Chinese Medical Association (2015). The guideline of prevention and treatment for chronic hepatitis B: a 2015 update. Zhonghua Gan Zang Bing Za Zhi.

[7] Shibayama T, Masuda G, Ajisawa A, Hiruma K, Tsuda F, Nishizawa T, Takahashi M, Okamoto H (2005). Characterization of seven genotypes (A to E, G and H) of hepatitis B virus recovered from Japanese patients infected with human immunodeficiency virus type 1. J Med Virol.

[8] Ghosh S, Banerjee P, Deny P, Mondal RK, Nandi M, Roychoudhury A, Das K, Banerjee S, Santra A, Zoulim F, Chowdhury A, Datta S (2013). New HBV subgenotype D9, a novel D/C recombinant, identified in patients with chronic HBeAg-negative infection in Eastern India. J Viral Hepat.

[9] Echevarría JM, Avellón A (2006). Hepatitis B virus genetic diversity. J Med Virol.

[10] Girones R, Miller RH (1989). Mutation rate of the hepadnavirus genome. Virology.

[11] Tipples GA, Ma MM, Fischer KP, Bain VG, Kneteman NM, Tyrrell DL (1996). Mutation in HBV RNA-dependent DNA polymerase confers resistance to lamivudine in vivo. Hepatology.

[12] Mackay IM, Arden KE, Nitsche A (2002). Real-time PCR in virology. Nucleic Acids Res.

[13] Allice T, Cerutti F, Pittaluga F, Varetto S, Gabella S, Marzano A, Franchello A, Colucci G, Ghisetti V (2007). COBAS AmpliPrep-COBAS TaqMan hepatitis B virus (HBV) test: a novel automated real-time PCR assay for quantification of HBV DNA in plasma. J Clin Microbiol.

[14] Lindh M, Hannoun C, Malmström S, Lindberg J, Norkrans G (2006). Lamivudine resistance of hepatitis B virus masked by coemergence of mutations in probe region of the COBAS AMPLICOR assay. J Clin Microbiol.

[15] Shah K, Ragupathy V, Saga A, Hewlett I (2016). High sensitivity detection of HIV-1 using two genomic targets compared with single target PCR. J Med Virol.

[16] Korn K, Weissbrich B, Henke-Gendo C, Heim A, Jauer CM, Taylor N, Eberle J (2009). Single-point mutations causing more than 100-fold underestimation of human immunodeficiency virus type 1 (HIV-1) load with the Cobas TaqMan HIV-1 real-time PCR assay. J Clin Microbiol.

[17] Sun S, Meng S, Zhang R, Zhang K, Wang L, Li J (2011). Development of a new duplex real-time polymerase chain reaction assay for hepatitis B viral DNA detection. Virol J.

[18] Liang X, Bi S, Yang W, Wang L, Cui G, Cui F, Zhang Y, Liu J, Gong X, Chen Y, Wang F, Zheng H, Wang F, Guo J, Jia Z, Ma J, Wang H, Luo H, Li L, Jin S, Hadler SC, Wang Y (2009). Epidemiological serosurvey of hepatitis B in China--declining HBV prevalence due to hepatitis B vaccination. Vaccine.

[19] Günther S, Li BC, Miska S, Krüger DH, Meisel H, Will H (1995). A novel method for efficient amplification of whole hepatitis B virus genomes permits rapid functional analysis and reveals deletion mutants in immunosuppressed patients. J Virol.

[20] Stuyver L, De Gendt S, Van Geyt C, Zoulim F, Fried M, Schinazi RF, Rossau R (2000). A new genotype of hepatitis B virus: complete genome and phylogenetic relatedness. J Gen Virol.

[21] Vermehren J, Kau A, Gärtner BC, Göbel R, Zeuzem S, Sarrazin C (2008). Differences between two real-time PCR-based hepatitis C virus (HCV) assays (RealTime HCV and Cobas AmpliPrep/Cobas TaqMan) and one signal amplification assay (Versant HCV RNA 3.0) for RNA detection and quantification. J Clin Microbiol.

[22] Chevaliez S, Bouvier-Alias M, Pawlotsky JM (2009). Performance of the Abbott real-time PCR assay using m2000sp and m2000rt for hepatitis C virus RNA quantification. J Clin Microbiol.

[23] Meng S, Li J (2010). A novel duplex real-time reverse transcriptase-polymerase chain reaction assay for the detection of hepatitis C viral RNA with armored RNA as internal control. Virol J.

[24] Huang J, Yang CM, Wang LN, Meng S, Deng W, Li JM (2008). A novel real-time multiplex reverse transcriptase-polymerase chain reaction for the detection of HIV-1 RNA by using dual-specific armored RNA as internal control. Intervirology.

[25] Andonov A, Osiowy C, Borlang J, Swidinsky K (2016). Sequence variability of the Cobas taqman assay target region impacts accurate HBV DNA detection. Vox Sang.

